# Sildenafil Citrate for Prophylaxis of Nephropathy in an Animal Model of Contrast-Induced Acute Kidney Injury

**DOI:** 10.1371/journal.pone.0113598

**Published:** 2014-11-26

**Authors:** D. Adam Lauver, E . Grant Carey, Ingrid L. Bergin, Benedict R. Lucchesi, Hitinder S. Gurm

**Affiliations:** 1 Department of Pharmacology, University of Michigan, Ann Arbor, MI, United States; 2 Unit for Laboratory Animal Medicine, University of Michigan, Ann Arbor, MI, United States; 3 Department of Cardiovascular Medicine, University of Michigan, Ann Arbor, MI, United States; School of Public Health of University of São Paulo, Brazil

## Abstract

Contrast-induced acute kidney injury (CIAKI) is one of the commonest complications associated with contrast media (CM). Although the exact etiology of CIAKI remains unclear, one hypothesis involves vasoconstriction of afferent arterioles resulting in renal ischemia. Increased renal blood flow, therefore, might represent an attractive target for the treatment of CIAKI. In this study we evaluated the protective effects of the phosphodiesterase type 5 (PDE5) inhibitor, sildenafil citrate, in a rabbit model of CIAKI. New Zealand white rabbits were used due to their susceptibility to CIAKI. To evaluate the effects of sildenafil, the drug was administered before CM infusion and repeatedly throughout the remainder of the experiment (6 mg/kg, p.o.). Animals were sacrificed after 48 hours and kidneys were prepared for histological evaluation. Intravenous administration of CM produced marked kidney injury. Serum creatinine concentrations were elevated within two hours of the infusion and remained elevated for the duration of the experiment. Histological evaluation of the kidneys revealed significant tubular necrosis. The effects of the CM were dose dependent. Treatment with sildenafil was associated with lesser degree of histological injury, attenuation in markers of acute kidney injury (48 hour creatinine 1.54±0.21 versus 4.42±1.31 mg/dl, p<0.05) and reduction in electrolyte derangement (percent change in serum K^+^ at 48 hours 2.55±3.80% versus 15.53±4.47%, p<0.05; serum Na^+^ at 48 hours −0.14±0.26% versus −1.97±1.29%, p = 0.20). The results suggest a possible role for PDE5 inhibitors in the treatment of CIAKI and warrant further evaluation to determine the exact mechanism of protection.

## Introduction

Contrast-induced acute kidney injury (CIAKI) is a complex syndrome of acute nephropathy occurring within 48 hours of exposure to intravascular iodinated contrast media (CM) [1], [2]. CIAKI is associated with an increased risk of adverse cardiovascular events, prolonged hospitalization, and short- and long-term mortality [3]–[5]. The pathophysiology of CIAKI is poorly understood and little is known about the underlying cellular mechanisms. With the increasing use of CM in both diagnostic and interventional procedures, CIAKI has become the third leading cause of hospital-acquired AKI, accounting for about 12% of the cases [6]. The incidence of CIAKI remains high despite the introduction of newer and safer contrast media and improved hydration protocols [7], [8].

Numerous *in vitro* and animal studies of CIAKI have contributed to knowledge of the condition [9]. The pathophysiology of CIAKI is hypothesized to involve both direct cellular toxicity and reduced renal blood flow resulting in localized renal ischemia. Several cell culture studies have demonstrated the direct cytotoxic effects of iodinated contrast agents on a variety of renal cell lines [10], [11]. The toxic effects of CM were partially prevented by the addition of antioxidant compounds suggesting a potential pathological role for free radicals [12]. Additionally, i*n vivo* studies using animal models of CIAKI have demonstrated prolonged constriction of the renal vasculature. The reduction in renal blood flow appeared to be constrained to specific areas of the kidney, namely the cortical and outer medullary regions [13]–[16]. More recently, magnetic resonance imaging studies have confirmed these findings and suggest that these techniques may be a sensitive diagnostic tool in the clinic [17], [18].

Pharmacological interventions against CIAKI have proven to be largely ineffective in the clinical setting and therefore the development of novel therapeutic interventions continues to be a topic of intense research interest [19], [20]. Current best treatment practices involve the administration of oral and intravenous fluids before, during, and after exposure to CM for the prevention kidney injury. This practice has a long history in clinical medicine and is currently the only treatment included in the guidelines of the American Heart Association, American College of Cardiology, the Society for Cardiovascular Angiography and Intervention and European Society of Urogenital Radiology [21], [22]. The rationale by which fluid administration is protective of the kidney is complex and includes reducing the urine concentration of CM, decreasing renal vasoconstrictive factors and diminishing renal oxygen consumption [23]–[27]. Despite the benefits of hydration therapy, the amounts, type and route of administration are not well established.

Based on the postulated pathophysiology of CIAKI, a favorable treatment target would be increasing renal blood flow through the induction of local vasodilation. Cyclic-GMP phosphodiesterase type 5 (PDE5) inhibitors have previously been demonstrated to reduce renal injury due to ischemia [28]–[30]. These agents block the degradation of cyclic GMP in vascular smooth muscle causing relaxation and vasodilation. Previous studies have demonstrated the vasodilatory action of these agents in the kidney [31], [32]. Moreover, a recent report suggests these agents can promote recovery from AKI through the induction of mitochondrial biogenesis (MB) [33]. Therefore we hypothesized that inhibition of PDE5 by the drug sildenafil citrate, will protect the kidneys from CIAKI. In this study, we used a rabbit model of CIAKI to evaluate the protective effects of sildenafil citrate. Renal function was measured using serum creatinine and plasma ion concentrations. In addition, pathological changes in the kidney were determined by histological evaluation. The results suggest that PDE5 inhibitors may be beneficial in the prevention of CIAKI.

## Methods

### Reagents

All chemicals and reagents were obtained from Sigma-Aldrich (St. Louis, MO) unless otherwise indicated. Sildenafil citrate (*Revatio*, Pfizer Inc., New York, NY) was purchased from the University of Michigan Ambulatory Pharmacy. Tablets were finely crushed and the powder was placed in gelatin capsules for oral administration to the rabbits. Ioxilan (*Oxilan 350*, Guerbet LLC, Bloomington, IN) was purchased from directly from the manufacturer. Ketamine HCl (*Ketaset*, Fort Dodge Animal Health, Fort Dodge, IA) and xylazine (*AnaSed*, Lloyd Inc., Shenandoah, IA) were purchased from Henry Schein Animal Health (Dublin, OH).

### Guidelines for animal research

The procedures used in this study have been approved by the University of Michigan University Committee on the Use and Care of Animals (Protocol No. PRO00004870) and conform to the standards in The Guide for Care and Use of Laboratory Animals (NIH No. 86–23). The University of Michigan Unit for Laboratory Animal Medicine provided veterinary care and is accredited by the American Association of Accreditation of Laboratory Animal Care.

### Rabbit Model of Contrast-Induced Nephropathy

Twenty-five male New Zealand white rabbits (weighing approximately 2.1–3.1 kg each) were randomly assigned to six treatment groups (n = 3–5 per group) and subjected to CIAKI. Animals were housed in a climate-controlled room and given free access to food and water throughout the study. The CIAKI model was developed on the basis of previously described rabbit renal contrast toxicity test [34] and involves intravenous administration of iodinated contrast agent (ioxilan) through the rabbit marginal ear vein over a period of 30 min. At the beginning of each experiment, the animals were weighed and anesthetized with a combination of ketamine (40 mg/kg, IM) and xylazine (5 mg/kg, IM). A cannula was placed in a marginal ear vein for administration on the contrast agent. A second cannula was inserted into a central ear artery for blood collection. Groups 1–4 (n = 4–5) animals received 2.5, 5.0, 7.5 and 10.0 g I/kg ioxilan IV, respectively; Group 5 (n = 3) animals received a gelatin capsule containing 6 mg/kg sildenafil citrate p.o. 30 minutes before infusion of 5.0 g I/kg ioxilan IV; and Group 6 (n = 5) animals received a gelatin capsule containing 6 mg/kg sildenafil citrate p.o. 30 minutes before infusion of 5.0 g I/kg ioxilan IV with additional sildenafil treatments occurring every 8 hours for the remainder of the study. Capsules were administered with the assistance of a rubber-tipped "pet piller" while the animals were loosely restrained. Blood samples were collected approximately 5 minutes before and then again at 1, 2, 24, and 48 hours after intravenous administration of contrast media. The volume of CM used in Groups 2, 5 and 6 was 14.3 ml/kg or approximately 3 times the estimated GFR [35]. This dose is in accordance with previously published clinical data from our group [36]. Rabbits were monitored for 48 hours after contrast infusion at which time they were euthanized (intravenous barbiturate overdose).

### Serum creatinine determination

Whole blood (3 ml per time point) was collected at various time points from the central ear artery and allowed to clot at room temperature in a glass test tube for 30 min. The tubes were then centrifuged at 1,500×g for 10 min at 4°C. The resulting supernatant was rapidly frozen in liquid nitrogen and stored at less than −70°C until the day of the assay. Serum creatinine concentrations were determined by the Michigan Diabetes Research and Training Center Chemistry Laboratory using a Multiplex assay (Luminex xMap, Luminex Corporation, Austin, TX) performed on a Milliplex Analyzer (EMD Millipore, Billerica, MA).

### Plasma ion measurement

Small samples (<1 ml) of whole blood were collected from the central ear artery into syringes containing 50 µl of unfractionated heparin (1000 U/ml). The blood samples were rapidly analyzed using a RapidLab 348 blood gas and ion analyzer (Siemens Healthcare, Deerfield, IL).

### Histological evaluation of kidneys

Kidneys were harvested and immediately fixed in 4% paraformaldehyde. Left kidneys were longitudinally sectioned and right kidneys were transversely sectioned. After fixation, tissues were embedded in paraffin using an automated histology processor following standard histological protocols. Replicate sections were cut at 5 µm thickness on a rotary microtome and stained with hematoxylin and eosin on an automated stainer. Histological sections were evaluated by a board-certified veterinary pathologist who was blinded to the experimental groups at the time of evaluation. Histological alterations were evaluated by a modification of a previously described scoring system for evaluation of CIAKI [37]. This consisted of assessment for tubular damage and protein casts according to the following key: 0, no alterations; 1, mild, isolated foci, <5% tubules affected; 2, moderate, 5–25% tubules affected; 3, marked, 26–50% tubules affected; 4, severe, >50% tubules affected. Tubular damage was defined as dilatation, degeneration, and/or necrosis, alterations which exist as a time and severity spectrum of tubular epithelial cell damage. Each parameter (tubular damage, protein casts) had a maximal individual score of 4. The individual scores were added to generate a summary score for renal damage (maximal score 8). Glomeruli were also assessed, but were not included as part of the scoring system since glomerular lesions are not part of the typically reported pathology of acute CIAKI models. Representative photomicrographs were taken using an Olympus DP72 12.5 megapixel digital camera mounted to an Olympus BX45 light microscope and using the manufacturer software (cellSens Standard 1.7.1, Olympus Corp.). Photo processing and composite plate construction was performed in Adobe Photoshop CS2, version 9.0. Photo processing was confined to global adjustments of brightness, contrast, sharpness, and image size, which did not materially alter the appearance, or interpretation of the image.

### Statistical analysis

The data are expressed as mean ± SEM of 3–5 experiments unless otherwise noted. One-way analysis of variance for repeated measurements followed by a Dunnett's post hoc t test to determine the level of significance was used to evaluate statistical differences for kidney to body weight ratio, serum creatinine concentration, plasma ion concentration, and kidney pathology scores. Values were considered to be statistically different at a level of p<0.05. Statistical analyses were performed using Prism software (GraphPad Software, La Jolla, CA).

## Results

### Kidney to body weight ratio

**Figure 1:**
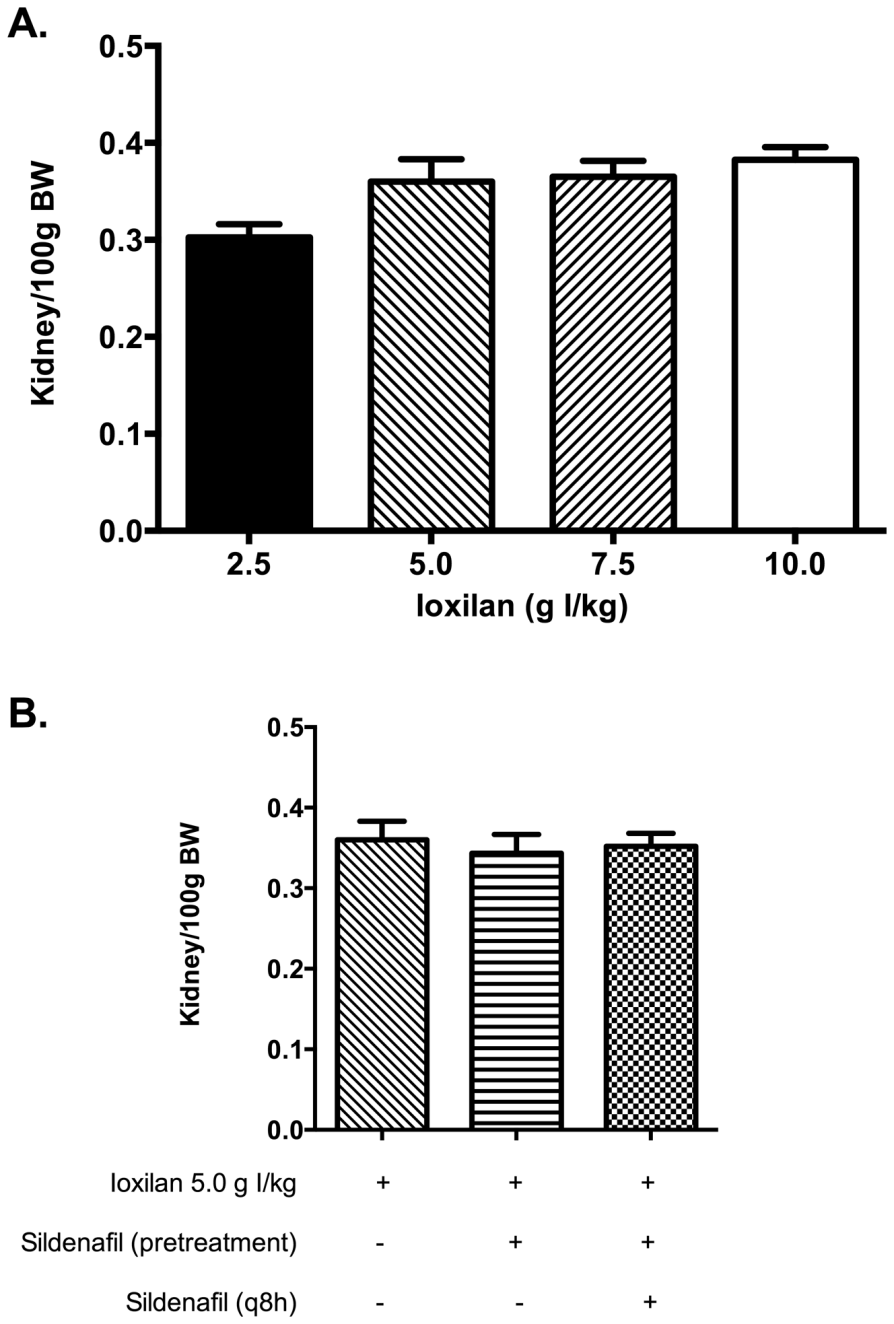
Kidney to body weight ratio analysis in rabbits subjected to CIAKI. The kidney to body weight ratio was measured as the average weight of both kidneys per 100 g body weight. (a) Kidney to body weight ratio for animals treated with increasing doses of CM. (b) Kidney to body weight ratio for animals pretreated with sildenafil citrate (6.0 mg/kg; single dose or q8 h).

At baseline there were no significant differences in mean body weight between treatment groups (data not shown). As illustrated in Figure 1, the administrations of contrast media caused minor elevations in kidney to body weight ratios. Simultaneous treatment with sildenafil citrate (6.0 mg/kg) resulted in an insignificant reduction in kidney to body weight ratios, regardless of dosing regimen.

### Serum creatinine concentration

**Figure 2:**
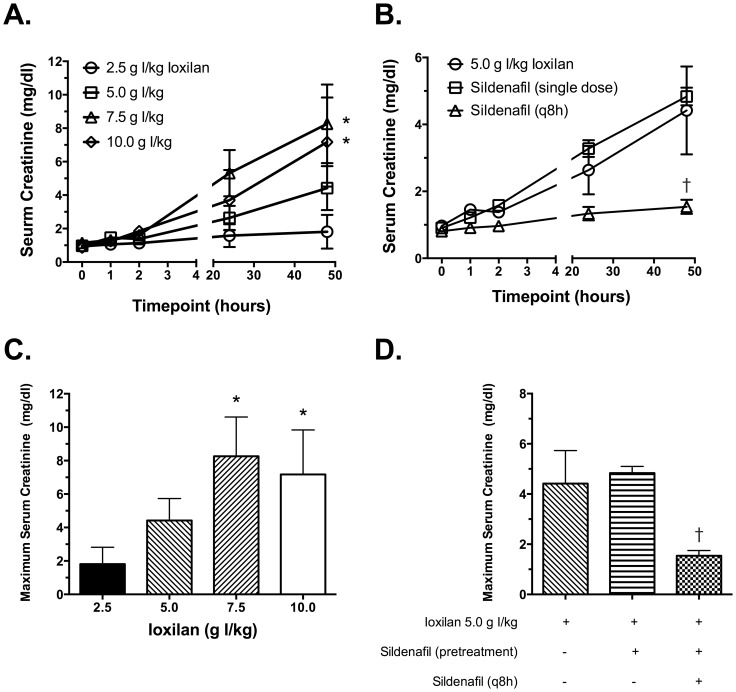
The effects of sildenafil citrate on CM-induced renal dysfunction. Serum samples were prepared from whole blood collected at various time points. Creatinine concentrations were determined using a Luminex xMap assay. (A) Time course of serum creatinine concentration in animals treated with increasing concentrations of CM. (B) Time course of serum creatinine concentration in animals pretreated with sildenafil citrate (6.0 mg/kg; single dose or q8 h) prior to CM exposure (5.0 g I/kg). (C) Maximum serum creatinine concentration in animals treated with increasing concentrations of CM. (D) Maximum serum creatinine concentration in animals pretreated with sildenafil citrate (6.0 mg/kg; single dose or q8 h) prior to CM exposure (5.0 g I/kg). Values are presented as mean ± SEM; n = 3–5. * indicates p<0.05 vs. 2.5 g I/kg and † indicates p<0.05 versus 5.0 g I/kg by one-way ANOVA and Dunnett's multiple comparisons test.

Serum creatinine concentration was measured as an indicator of kidney function. At baseline serum creatinine were similar in all treatment groups. As demonstrated in Figure 2A, serum creatinine concentrations began to increase between one and two hours after the end of CM infusion. The increase in serum creatinine was highly dependent on the CM dose. In most animals, maximal serum creatinine concentration was observed at 48 hours; hence this time point was used to compare maximal serum creatinine concentrations (Figure 2B). Continuous sildenafil treatment (6.0 mg/kg; q8 h) significantly attenuated the rise in serum creatinine concentration (p<0.05), whereas a single dose of sildenafil before CM exposure had no effect (Figure 2C and 2D).

### Plasma ion concentrations

**Figure 3:**
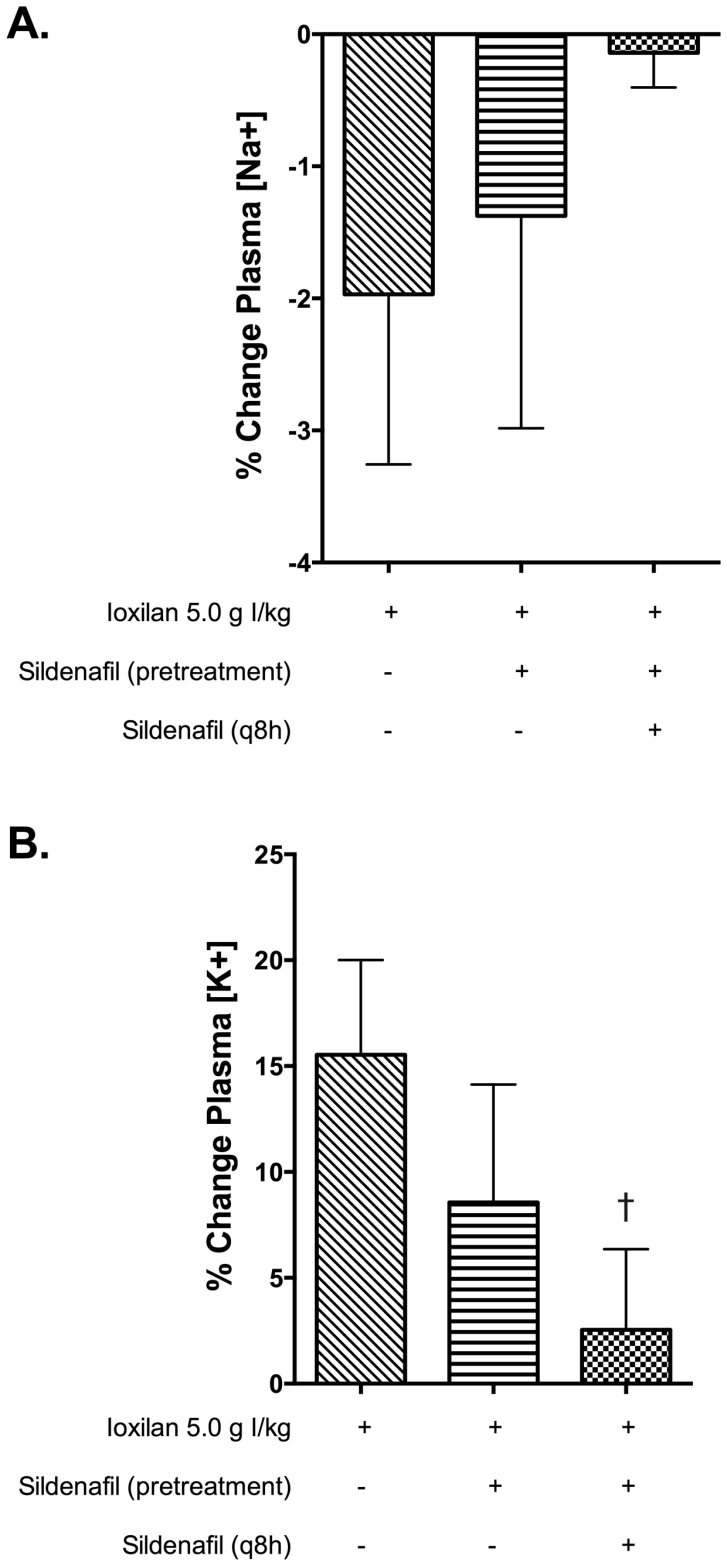
Repeated treatment with sildenafil citrate prevents hyponatremia and hyperkalemia in animals exposed to CM. (A) Percent change (t = 0 vs. 48 h) in plasma Na^+^ concentrations in animals administered intravenous ioxilan (5.0 g I/kg) with/without simultaneous sildenafil therapy. (B) Percent change (t = 0 vs. 48 h) in plasma K^+^ concentrations in animals administered intravenous ioxilan (5.0 g I/kg) with/without simultaneous sildenafil therapy. Values are presented as mean ± SEM; n = 3–5. † indicates p<0.05 versus 5.0 g I/kg by one-way ANOVA and Dunnett's multiple comparisons test.

Plasma concentrations of Na^+^ and K^+^ were determined at baseline and immediately prior to sacrifice as a measure of kidney function in animals exposed to CM. At baseline Na^+^ and K^+^ concentrations were similar in all cohorts (data not shown). As illustrated in Figure 3, exposure to CM (ioxilan, 5.0 g I/kg) elicited a reduction in plasma Na^+^ concentration and an increase in plasma K^+^ concentration at the time of sacrifice (48 hours post exposure). Continuous treatment of sildenafil citrate (6.0 mg/kg), but not a single pretreatment dose, diminished the observed changes in Na^+^ and K^+^ concentration (p<0.05).

### Histological evaluation of kidney sections

**Figure 4:**
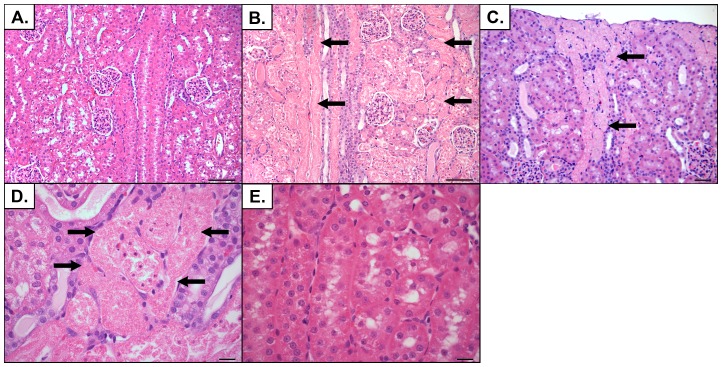
Representative histological lesions in kidneys from rabbits subjected to CIAKI. Renal cortex from rabbits treated with (A) 2.5 g I/kg CM, (B) 10.0 g I/kg CM, or (C) 5.0 g I/kg CM and sildenafil citrate (6.0 mg/kg, q8 h). In a rabbit treated with 2.5 g I/kg there were no histological alterations in the cortex. (A, summary pathology score  = 0) In a rabbit treated with 10.0 g I/kg there was severe tubular necrosis (arrows) affecting >50% of the cortical tubules. (B, summary pathology score  = 7). In a rabbit treated with 5.0 g I/kg CM and sildenafil citrate there was tubular necrosis affecting a much smaller percentage of cortical tubules (C, summary pathology score  = 3). Higher magnification of tubular necrosis from a rabbit in the 10.0 g I/kg group shows details of tubular necrosis, (arrows) evidenced by loss of cellular detail in the tubules, nuclear loss or condensation, and sloughing of necrotic cell debris into the tubule lumen. (D). A similar area in a rabbit treated with 2.5 g I/kg CM shows no alterations in the tubules (E). Hematoxylin and eosin stain. Original magnifications for A, B, C = ×200; bars  = 100 µm; for D, E  = ×600, bar  = 20 µm.

**Figure 5:**
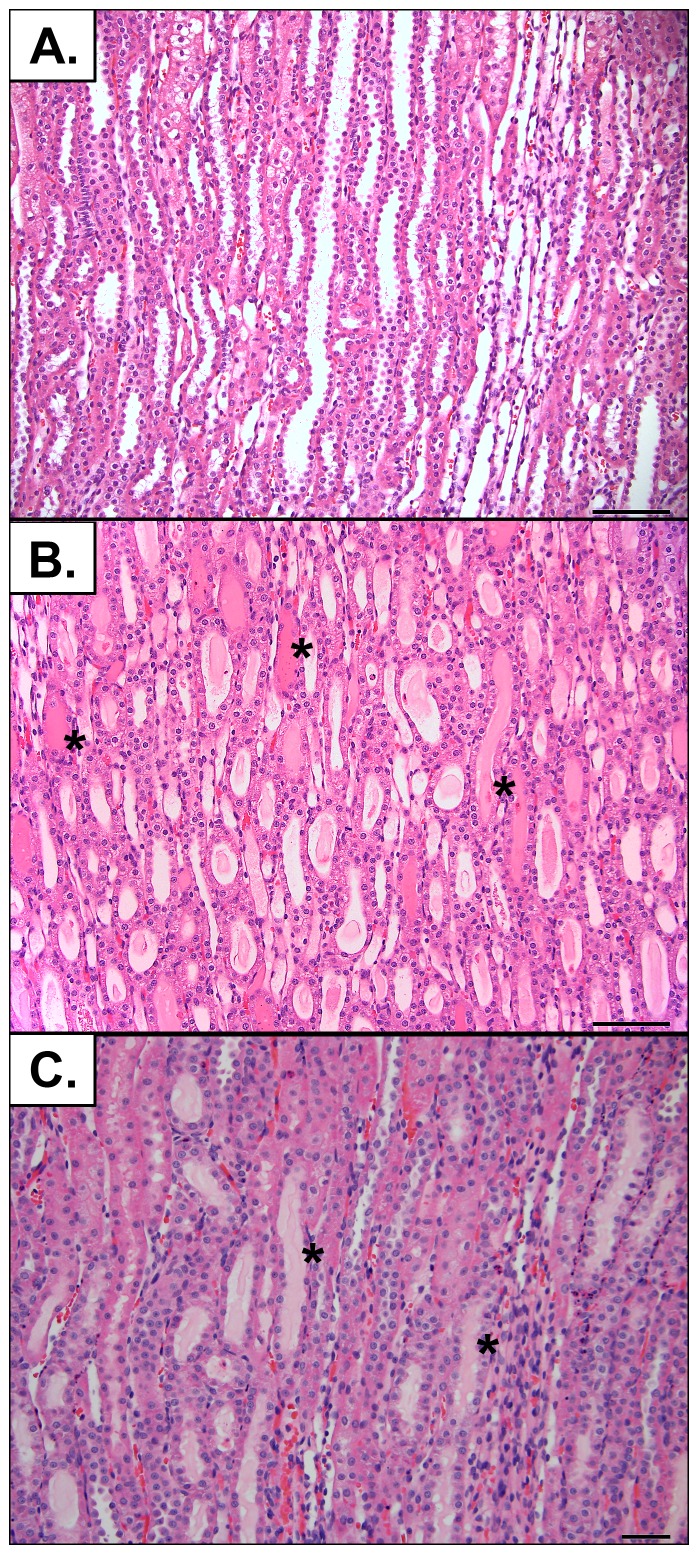
Representative histological lesions in kidneys from rabbits subjected to CIAKI. Outer stripe of the renal medulla from rabbits treated with (A) 2.5 g I/kg CM, (B) 10.0 g I/kg CM, or (C) 5.0 g I/kg CM and sildenafil citrate (6.0 mg/kg, q8 h). Damage consisted of intraluminal pale eosinophilic (proteinaceous) exudate. Hematoxylin and eosin stain. Original magnification ×200; bars  = 100 µm.

**Figure 6:**
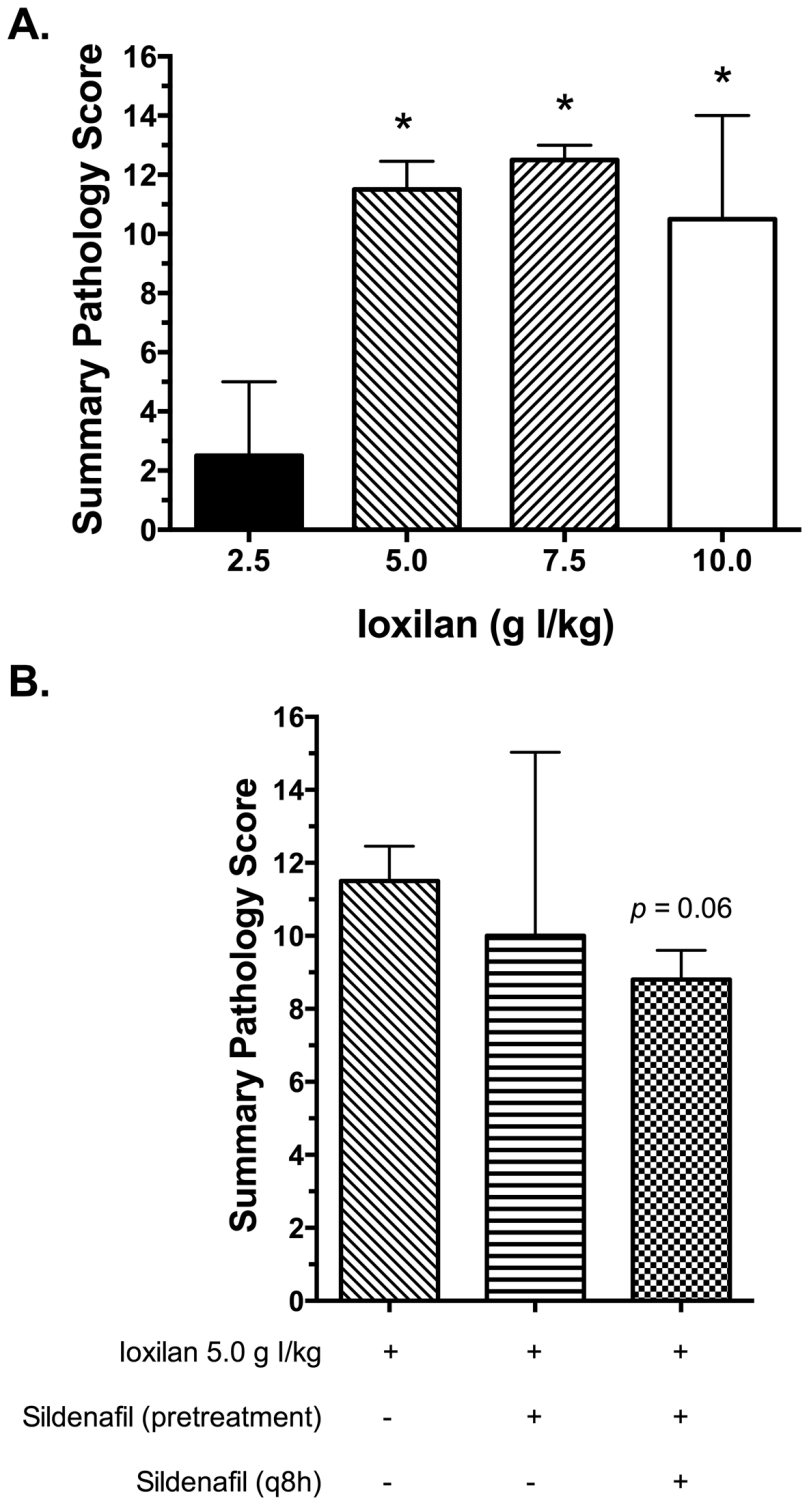
Kidney pathology scores from rabbits subjected to CIAKI. Summary pathology scores were calculated as outlined in the methods. (A) Pathology scores for animals treated with increasing doses of CM. (B) Pathology scores for animals pretreated with sildenafil citrate (6.0 mg/kg; single dose or q8 h) prior to CM exposure (5.0 g I/kg). Values are presented as mean ± SEM; n = 3–5. * indicates p<0.05 vs. 2.5 g I/kg.

Representative images from the cortical and medullary regions of the kidneys are shown in Figures 4 and 5, respectively. The severity of histological changes was dependent upon the dose of CM (Figure 6A). The predominant change was tubular necrosis. For the less severely affected rabbits, tubular necrosis was multifocally distributed and confined to the outer cortex (S1 portion of the proximal tubule). For the more severely affected rabbits, tubular necrosis extended deeper into the cortex with fewer intervening normal nephrons. Histological lesion severity was blunted by continuous treatment with sildenafil citrate (p = 0.06), but not by a single pretreatment dose (Figure 6B). The principal lesions observed in the kidneys consisted of tubular necrosis and predominantly affected the proximal tubules in the cortex (Figure 4). Animals with higher histological scores had a greater percent of cortical tubular involvement and extension of the lesion deeper into the cortex and outer portion of the medulla. Animals with lower histological scores had a smaller percent involvement of cortical tubules without significant extension of the lesion into the deeper cortical or medullary regions. Although tubular necrosis was the predominant finding, tubular degeneration (evidenced by cell swelling and vacuolation) was also present adjacent to areas of necrosis in some tubules. Luminal protein casts were also present to a limited degree in the outer medulla. Glomerular changes were not present.

## Discussion

The present study provides the first evidence that sildenafil citrate has therapeutic potential in the prevention and possibly treatment of CIAKI by attenuating renal injury and preserving renal function in an experimental rabbit model. We demonstrate the dose dependent toxic effects of an iodinated contrast agent (*Oxilan*, ioxilan) in New Zealand white rabbits. The model was developed based on a previous report by Pettersson and colleagues investigating the susceptibility of rabbits to various iodinated contrast media [34]. In this study, serum creatinine concentrations were rapidly elevated following exposure to intravenous CM. Creatinine concentrations continued to increase until the time of sacrifice and were positively correlated with the dose of CM. Similarly, histological evaluation of kidneys post mortem indicated extensive tubular necrosis and epithelial disruption. Taken together these findings indicate severe renal dysfunction and injury. Kidney to body weight ratios, however, were largely unchanged suggesting insignificant tissue inflammation and edema during this timespan. Based on these initial findings, 5.0 g I/kg CM was chosen to test the potential protective effect of sildenafil. The dose was chosen both on the empirical evidence from dose response and also because this dose closely reflects CM exposure in clinical practice and induced similar increases in serum creatinine concentration [36], [38].

The deleterious effects of CM were abrogated by repeated oral administrations (6.0 mg/kg, every 8 hours) of sildenafil. Cyclic-GMP phosphodiesterase type 5 inhibitors, like sildenafil, represent an attractive class of compounds for the treatment of CIAKI for a variety of reasons. First, these compounds have been shown to reduce ischemic injury to the kidney and improve local renal blood flow [28]–[32]. In addition, this therapeutic class has been used clinically for more than two decades for the treatment of erectile dysfunction and pulmonary hypertension and in this time have proven to be safe and void of serious side effects [39].

We observed that repeated treatment with sildenafil blunted the increase in serum creatine concentration. Additional, hyponatremia and hyperkalemia, which are hallmarks of acute renal injury, were blunted by repeated administration of sildenafil citrate. From a histological viewpoint, the deleterious effects of CM on the kidney were also reduced in animals repeatedly administered sildenafil. In this study, tubular necrosis was more common than tubular degeneration or dilatation. Interestingly, sildenafil treatment did not reduce the pathological changes in the kidney to the same degree as the changes in serum creatinine concentration. This finding suggests that the drug may act, in part, by improving the function of the remaining nephrons rather than preventing direct renal injury due to CM exposure.

Restoration of mitochondrial number and function is known to be an important factor in recovery from AKI. In addition to its vasodilatory effects, another potential protective mechanism of sildenafil might be stimulation of MB. A recent report by Whitaker and colleagues demonstrated the stimulatory effects of sildenafil on MB in the kidney [33]. Sildenafil exerts this effect by potentiating the effects of cGMP thereby increasing the expression of peroxisome proliferator-activated receptor γ coactivator 1α (PGC-1α). PGC-1α activates a series of downstream mediators, which ultimately increases the transcription of mitochondrial DNA leading to MB. Further study is warranted to further elucidate the exact protective mechanism of sildenafil.

Linkermann and colleagues have recently implicated necroptosis in the pathophysiology of CIAKI [40]. Morphologically, necroptosis is identical to “classical” necrosis, which is histologically defined as affecting sheets of adjacent cells with the alterations of cell swelling, hypereosinophilia, and nuclear pyknosis, karyorrhexis, or karyolysis. Necroptosis is distinguished from necrosis principally by the involvement of the RIP complex and cannot be determined by light microscopy. Therefore, we are unable to distinguish the manner of the cell death in our study and the question remains as to whether sildenafil might act by interfering the necroptotic pathway.

Importantly, the protective effects of sildenafil were conferred only when the drug was given repeatedly (every 8 hours) and not as a single pretreatment. This observation may be due, in part, to the relatively short half-life of sildenafil (4 hours) and the prolonged constriction of the renal vasculature (24–48 hours) following administration of CM [18], [41]. Another PDE5 inhibitor, tadalafil, has a much longer half-life (17.5 hours) and could find increased utility in this setting due to the reduced dosing frequency [42].

Clinically, the incidence of CIAKI is greater in patients with preexisting renal insufficiency [6], [43]. It should be noted, however, that our animal model of CIAKI used rabbits with normal kidney function. To further complicate this situation, the kidneys are partially responsible for the excretion of sildenafil and its active metabolite [44]. As a consequence, repeated treatment in compromised patients could result in accumulation of the drug and represent a significant concern for toxicity and hypotension, especially in those patients with severe renal dysfunction [45]. As a consequence the question remains of whether sildenafil treatment will afford a similar risk-benefit ratio in clinical settings. It is likely that the drug will be safe since in prior human trials designed to access the efficacy of sildenafil in diabetic patients and those with renal dysfunction, the actions of the drug were minimally affected by these comorbidities [46]–[48].

Our results indicate that sildenafil citrate, when given repeatedly during the first 48 hours following CM exposure, is capable of protecting the renal tubules and preserving normal kidney function. PDE5 inhibitors, like sildenafil, represent an attractive class of agents that may be a useful therapeutic addition to existing hydration procedures. Further study is warranted to determine the optimal dose and frequency in humans for the prevention and treatment of CIAKI.

## References

[1] Finn WF (2006) The clinical and renal consequences of contrast-induced nephropathy. Nephrol Dial Transplant 21:i2–10. 16723349 10.1093/ndt/gfl213

[2] McCullough PA, Soman SS (2005) Contrast-induced nephropathy. Crit Care Clin 21:261–280. 15781162 10.1016/j.ccc.2004.12.003

[3] McCullough PA, Wolyn R, Rocher LL, Levin RN, O'Neill WW (1997) Acute renal failure after coronary intervention: incidence, risk factors, and relationship to mortality. Am J Med 103:368–375. 9375704 10.1016/s0002-9343(97)00150-2

[4] Rihal CS, Textor SC, Grill DE, Berger PB, Ting HH, et al (2002) Incidence and prognostic importance of acute renal failure after percutaneous coronary intervention. Circulation 105:2259–2264. 12010907 10.1161/01.cir.0000016043.87291.33

[5] Best PJ, Lennon R, Ting HH, Bell MR, Rihal CS, et al (2002) The impact of renal insufficiency on clinical outcomes in patients undergoing percutaneous coronary interventions. J Am Coll Cardiol 39:1113–1119. 11923033 10.1016/s0735-1097(02)01745-x

[6] McCullough PA, Adam A, Becker CR, Davidson C, Lameire N, et al (2006) Epidemiology and prognostic implications of contrast-induced nephropathy. Am J Cardiol 98:5K–13K. 10.1016/j.amjcard.2006.01.019 16949375

[7] Maioli M, Toso A, Leoncini M, Micheletti C, Bellandi F (2011) Effects of hydration in contrast-induced acute kidney injury after primary angioplasty: a randomized, controlled trial. Circ Cardiovasc Interv 4:456–462. 21972403 10.1161/CIRCINTERVENTIONS.111.961391

[8] Bolognese L, Falsini G, Schwenke C, Grotti S, Limbruno U, et al (2012) Impact of iso-osmolar versus low-osmolar contrast agents on contrast-induced nephropathy and tissue reperfusion in unselected patients with ST-segment elevation myocardial infarction undergoing primary percutaneous coronary intervention (from the Contrast Media and Nephrotoxicity Following Primary Angioplasty for Acute Myocardial Infarction [CONTRAST-AMI] Trial). Am J Cardiol 109:67–74. 21943940 10.1016/j.amjcard.2011.08.006

[9] Tumlin J, Stacul F, Adam A, Becker CR, Davidson C, et al (2006) Pathophysiology of contrast-induced nephropathy. Am J Cardiol 98:14K–20K. 16949376 10.1016/j.amjcard.2006.01.020

[10] Andersen KJ, Christensen EI, Vik H (1994) Effects of iodinated x-ray contrast media on renal epithelial cells in culture. Invest Radiol 29:955–962. 7890510 10.1097/00004424-199411000-00002

[11] Heinrich MC, Kuhlmann MK, Grgic A, Heckmann M, Kramann B, et al (2005) Cytotoxic effects of ionic high-osmolar, nonionic monomeric, and nonionic iso-osmolar dimeric iodinated contrast media on renal tubular cells in vitro. Radiology 235:843–849. 15845795 10.1148/radiol.2353040726

[12] Romano G, Briguori C, Quintavalle C, Zanca C, Rivera NV, et al (2008) Contrast agents and renal cell apoptosis. Eur Heart J 29:2569–2576. 18468994 10.1093/eurheartj/ehn197

[13] Bakris GL, Burnett JC Jr. (1985) A role for calcium in radiocontrast-induced reductions in renal hemodynamics. Kidney Int 27:465–468. 2581011 10.1038/ki.1985.32

[14] Heyman SN, Brezis M, Epstein FH, Spokes K, Silva P, et al (1991) Early renal medullary hypoxic injury from radiocontrast and indomethacin. Kidney Int 40:632–642. 1745012 10.1038/ki.1991.255

[15] Liss P, Nygren A, Olsson U, Ulfendahl HR, Erikson U (1996) Effects of contrast media and mannitol on renal medullary blood flow and red cell aggregation in the rat kidney. Kidney Int 49:1268–1275. 8731090 10.1038/ki.1996.181

[16] Nygren A (1992) Contrast media and regional renal blood flow. A study of the effects of ionic and non-ionic monomeric and dimeric contrast media in the rat. Acta Radiol Suppl 378 (Pt 3):123–135. 1323194

[17] Zhang YD, Wang J, Zhang J, Wang X, Jiang X (2014) Effect of iodinated contrast media on renal function evaluated with dynamic three-dimensional MR renography. Radiology 270:409–415. 24091357 10.1148/radiol.13122495

[18] Zhang Y, Wang J, Yang X, Wang X, Zhang J, et al (2012) The serial effect of iodinated contrast media on renal hemodynamics and oxygenation as evaluated by ASL and BOLD MRI. Contrast Media Mol Imaging 7:418–425. 22649048 10.1002/cmmi.1468

[19] Chatterjee PK (2007) Novel pharmacological approaches to the treatment of renal ischemia-reperfusion injury: a comprehensive review. Naunyn Schmiedebergs Arch Pharmacol 376:1–43. 18038125 10.1007/s00210-007-0183-5

[20] Briguori C, Marenzi G (2006) Contrast-induced nephropathy: pharmacological prophylaxis. Kidney Int Suppl: S30–38. 16612399 10.1038/sj.ki.5000372

[21] Levine GN, Bates ER, Blankenship JC, Bailey SR, Bittl JA, et al (2011) 2011 ACCF/AHA/SCAI Guideline for Percutaneous Coronary Intervention. A report of the American College of Cardiology Foundation/American Heart Association Task Force on Practice Guidelines and the Society for Cardiovascular Angiography and Interventions. J Am Coll Cardiol 58:e44–122. 22070834 10.1016/j.jacc.2011.08.007

[22] Stacul F, van der Molen AJ, Reimer P, Webb JA, Thomsen HS, et al (2011) Contrast induced nephropathy: updated ESUR Contrast Media Safety Committee guidelines. Eur Radiol 21:2527–2541. 21866433 10.1007/s00330-011-2225-0

[23] Franchini KG, Mattson DL, Cowley AW Jr. (1997) Vasopressin modulation of medullary blood flow and pressure-natriuresis-diuresis in the decerebrated rat. Am J Physiol 272:R1472–1479. 9176339 10.1152/ajpregu.1997.272.5.R1472

[24] Larson TS, Hudson K, Mertz JI, Romero JC, Knox FG (1983) Renal vasoconstrictive response to contrast medium. The role of sodium balance and the renin-angiotensin system. J Lab Clin Med 101:385–391. 6338138

[25] Persson PB, Patzak A (2005) Renal haemodynamic alterations in contrast medium-induced nephropathy and the benefit of hydration. Nephrol Dial Transplant 20 Suppl 1i2–5. 15705945 10.1093/ndt/gfh1066

[26] Erley CM, Heyne N, Rossmeier S, Vogel T, Risler T, et al (1998) Adenosine and extracellular volume in radiocontrast media-induced nephropathy. Kidney Int Suppl 67: S192–194. 9736287 10.1046/j.1523-1755.1998.06744.x

[27] Tumkur SM, Vu AT, Li LP, Pierchala L, Prasad PV (2006) Evaluation of intra-renal oxygenation during water diuresis: a time-resolved study using BOLD MRI. Kidney Int 70:139–143. 16572109 10.1038/sj.ki.5000347 PMC2919062

[28] Kucuk A, Yucel M, Erkasap N, Tosun M, Koken T, et al (2012) The effects of PDE5 inhibitory drugs on renal ischemia/reperfusion injury in rats. Mol Biol Rep 39:9775–9782. 22736111 10.1007/s11033-012-1843-1

[29] Medeiros PJ, Villarim Neto A, Lima FP, Azevedo IM, Leao LR, et al (2010) Effect of sildenafil in renal ischemia/reperfusion injury in rats. Acta Cir Bras 25:490–495. 21120279 10.1590/s0102-86502010000600006

[30] Choi DE, Jeong JY, Lim BJ, Chung S, Chang YK, et al (2009) Pretreatment of sildenafil attenuates ischemia-reperfusion renal injury in rats. Am J Physiol Renal Physiol 297:F362–370. 19474186 10.1152/ajprenal.90609.2008

[31] Lledo-Garcia E, Subira-Rios D, Ogaya-Pinies G, Tejedor-Jorge A, Canizo-Lopez JF, et al (2011) Intravenous sildenafil as a preconditioning drug against hemodynamic consequences of warm ischemia-reperfusion on the kidney. J Urol 186:331–333. 21600605 10.1016/j.juro.2011.03.036

[32] Malavaud B, Rostaing L, Tran-Van T, Tack I, Ader JL (2001) Transient renal effects of sildenafil in male kidney transplant recipients. Transplantation 72:1331–1333. 11602866 10.1097/00007890-200110150-00027

[33] Whitaker RM, Wills LP, Stallons LJ, Schnellmann RG (2013) cGMP-selective phosphodiesterase inhibitors stimulate mitochondrial biogenesis and promote recovery from acute kidney injury. J Pharmacol Exp Ther 347:626–634. 24042162 10.1124/jpet.113.208017 PMC3836317

[34] Pettersson G, Towart R, Grant D, Thyberg K, Golman K (2002) The rabbit renal toxicity test: a sensitive in vivo test for the nephrotoxicity of contrast agents. Acad Radiol 9 Suppl 1S62–64. 12019897 10.1016/s1076-6332(03)80398-7

[35] Vadstrup S, Bojsen J (1983) The glomerular filtration rate in unrestrained rabbits determined by means of an implanted telemetrical device. Acta Physiol Scand 117:177–182. 6408892 10.1111/j.1748-1716.1983.tb07195.x

[36] Gurm HS, Dixon SR, Smith DE, Share D, Lalonde T, et al (2011) Renal function-based contrast dosing to define safe limits of radiographic contrast media in patients undergoing percutaneous coronary interventions. J Am Coll Cardiol 58:907–914. 21851878 10.1016/j.jacc.2011.05.023

[37] Ari E, Kedrah AE, Alahdab Y, Bulut G, Eren Z, et al (2012) Antioxidant and renoprotective effects of paricalcitol on experimental contrast-induced nephropathy model. Br J Radiol 85:1038–1043. 22815410 10.1259/bjr/16327485 PMC3587068

[38] Madsen TE, Pearson RR, Muhlestein JB, Lappe DL, Bair TL, et al (2009) Risk of nephropathy is not increased by the administration of larger volume of contrast during coronary angiography. Crit Pathw Cardiol 8:167–171. 19952552 10.1097/HPC.0b013e3181bda03b

[39] Leoni LA, Leite GS, Wichi RB, Rodrigues B (2013) Sildenafil: two decades of benefits or risks? Aging Male 16:85–91. 23758451 10.3109/13685538.2013.801952

[40] Linkermann A, Heller JO, Prokai A, Weinberg JM, De Zen F, et al (2013) The RIP1-kinase inhibitor necrostatin-1 prevents osmotic nephrosis and contrast-induced AKI in mice. J Am Soc Nephrol 24:1545–1557. 23833261 10.1681/ASN.2012121169 PMC3785275

[41] Nichols DJ, Muirhead GJ, Harness JA (2002) Pharmacokinetics of sildenafil after single oral doses in healthy male subjects: absolute bioavailability, food effects and dose proportionality. Br J Clin Pharmacol 53 Suppl 15S–12S. 11879254 10.1046/j.0306-5251.2001.00027.x PMC1874258

[42] Porst H, Padma-Nathan H, Giuliano F, Anglin G, Varanese L, et al (2003) Efficacy of tadalafil for the treatment of erectile dysfunction at 24 and 36 hours after dosing: a randomized controlled trial. Urology 62:121–125 discussion 125–126. 12837435 10.1016/s0090-4295(03)00359-5

[43] Lameire N, Adam A, Becker CR, Davidson C, McCullough PA, et al (2006) Baseline renal function screening. Am J Cardiol 98:21K–26K. 10.1016/j.amjcard.2006.01.021 16949377

[44] Wright PJ (2006) Comparison of phosphodiesterase type 5 (PDE5) inhibitors. Int J Clin Pract 60:967–975. 16780568 10.1111/j.1742-1241.2006.01049.x

[45] Wada Y, Kikuchi K, Imamura T (2009) A fatal hypotension by sildenafil in an end-stage renal disease patient with hypertension and abnormal pharmacokinetics of the medicine. Nephrology (Carlton) 14:357–358. 19426360 10.1111/j.1440-1797.2009.01132.x

[46] El-Sakka AI (2004) Efficacy of sildenafil citrate in treatment of erectile dysfunction: effect of type 2 diabetes. Eur Urol 46:503–509. 15363568 10.1016/j.eururo.2004.06.005

[47] Stuckey BG, Jadzinsky MN, Murphy LJ, Montorsi F, Kadioglu A, et al (2003) Sildenafil citrate for treatment of erectile dysfunction in men with type 1 diabetes: results of a randomized controlled trial. Diabetes Care 26:279–284. 12547849 10.2337/diacare.26.2.279

[48] Grossman EB, Swan SK, Muirhead GJ, Gaffney M, Chung M, et al (2004) The pharmacokinetics and hemodynamics of sildenafil citrate in male hemodialysis patients. Kidney Int 66:367–374. 15200445 10.1111/j.1523-1755.2004.00739.x

